# Awareness of the Consumption of Dietary Supplements among Students in a University in Saudi Arabia

**DOI:** 10.1155/2019/4641768

**Published:** 2019-05-02

**Authors:** Jozaa Z. AlTamimi

**Affiliations:** Assistant Professor of General Nutrition, Princess Nourah Bint Abdulrahman University, Riyadh 84428, Saudi Arabia

## Abstract

The dietary supplement market in Saudi Arabia is growing alongside the number of Saudis consuming dietary supplements. It is therefore increasingly important for healthcare personnel to understand the consumption rate of dietary supplements, whether they are used wisely, and the know-how concerning their use and dosage. The purpose of this study was to clarify the overall awareness of dietary supplement consumption among female students of Princess Nourah Bint Abdulrahman University, Riyadh, Saudi Arabia. Using a descriptive approach, data were collected from 759 female students (mean age = 22.1 ± 1.7 years) via an electronic survey. All participants reported having consumed dietary supplements at some point, and 32.3% were taking them at the time of the survey. They took supplements primarily for aesthetic reasons (e.g., hair and skin health) and preferred purchasing supplements at pharmacies. Nearly all (89.9%) participants could define supplements correctly, and 43.6% reported that supplements improved health. More than half the sample believed that supplements are safe. While a large proportion of participants correctly identified the recommended number of daily servings of fruits and vegetables, relatively few actually consumed that recommended amount. More than one-third of participants consumed more than one type of supplement simultaneously, and more than half reported that they would use supplements when necessary in the future. The results of this study suggest a need for long-term studies on the consumption of nutritional supplements among Saudis and its impact on health.

## 1. Introduction

Dietary supplements are defined as any product intended to supplement the diet and that contains one or more dietary ingredients [1]. They may include vitamins, minerals, herbs, meal supplements, and products that enhance nutrient levels [2, 3]. People consume nutritional supplements for many reasons, such as protecting against disease or health problems (e.g., stress, colds, heart attacks, osteoporosis, cancer, tooth decay, and neural tube defects in infants), increasing energy, improving physical performance, and correcting various lifestyle deficiencies [4–6]. As such, there are a number of types of supplements, such as those that compensate for inadequate daily dietary intake, those that help with accelerate weight loss, and those that improve the ability to gain weight and muscle [7–9].

In the United States, approximately 68% of adults use dietary supplements. However, in 2013, Asia and the Pacific was the largest food supplement market, accounting for 31.2% of the global market (followed by Europe and North America, at 30.1% and 25.4%, respectively). Between 2014 and 2020, the food supplement market in Asia and the Pacific is expected to show 9.1% growth [10]. In the Middle East, particularly in the Gulf states, the demand for dietary and herbal supplements is increasing [11–14]. In Saudi Arabia, the country's market growth is expanding with the rapid population growth, and the food supplements market now accounts for about 4% of total pharmaceutical market sales (roughly $80 million) [15]. Saudi Arabia is expected to see SR 875 million in sales of dietary supplements by 2021. This may be partly attributed to the fact that, in recent years, Saudis have increased their focus on the importance of maintaining health and protection from diseases [16]. Many students and athletes in Saudi Arabia and the Middle East as a whole consume dietary supplements and view them positively. The actual effectiveness and safety of these products might in fact arise from this belief that supplements are harmless and safe to use. However, the consumption of high quantities can have severe negative consequences: for example, high doses of vitamin D can lead to osteoporosis and muscle weakness [17–20]. Many supplements also contain active ingredients that have strong effects on the body and that might cause side effects, particularly when taken instead of prescribed drugs or when taken together with several other types of supplements [21]. In a study conducted at King Abdul Aziz Hospital in Jeddah, the prevalence of use of dietary supplements among a sample of patients was 22% (24% of whom were female) [22]. The prevalence of use was even higher, at 44.6%, among students of the Faculty of Medicine at Imam Abdulrahman Bin Faisal University in Dammam, Saudi Arabia [23], and higher still (76.6%) among female college students of King Saud University, 36.7% of whom did not know anything of the side effects of these products [24].

A potential problem related with the use of dietary supplements is that users might see them as a substitute, rather than a supplement, to the intake of fruits and vegetables. One study, conducted in Cape Town, South Africa, showed that a high proportion of participants who used supplements also consumed fewer vegetables and fruits, which are a rich source of vitamins and minerals, despite their awareness of the recommended quotas of vegetables and fruits [25]. Similar deficiencies in the intake of vegetables and fruits were found in Saudi Arabia among university students, with nearly 78% of a sample from King Faisal University in Al-Ahsa consuming fewer than 5 daily servings of vegetables and fruits [26]. Students of Dammam University in Saudi Arabia also consumed less than the recommended daily portions of vegetables and fruits, despite knowledge of the benefits [27].

The objective of this study was to determine the awareness of the use of dietary supplements among students of Princess Nourah Bint Abdulrahman University, Riyadh. Despite the large market for supplements in Saudi Arabia, there is little documentation of the prevalence of their use, especially among university students. The present study is particularly significant for its examination of the prevalence of dietary supplements among students of the largest female-only university in the world. This focus on females provides a glimpse at the awareness of supplement use among future mothers. As a secondary purpose, students' awareness of the recommended daily quotas of vegetables and fruits, and whether students actually met these quotas, was examined. As noted earlier, dietary supplements might cause problems because users see them as a substitute for fruits and vegetables. No study has yet explored both the use of dietary supplements and the consumption of vegetables and fruits in Saudi Arabia.

## 2. Methods

This was a descriptive, cross-sectional study. Prior to collecting any data, a pilot study was conducted with 30 female students who gave their approval to verify and validate the questionnaire. Specifically, tests of honesty and consistency were conducted to verify the validity and stability of the questionnaire. Permission to conduct the study was obtained from the Deanship of Scientific Research at the university. All participants were students of Princess Nourah Bint Abdulrahman University in Riyadh. Students were recruited by faculty members: specifically, a questionnaire link was sent by the Deanship of Scientific Research to faculty members for distribution among students, after obtaining students' informed consent. The consent procedure involved informing students of the objective of the study and their rights to confidentiality and to accept or refuse participation of their own volition. A total of 773 responses were obtained, 14 of which were excluded because they provided incomplete questionnaires. Thus, the data of 759 participants were analyzed (mean age = 22.1 ± 1.7 years). The questionnaire was sent at the beginning of 2017 and data collection continued throughout the 2017 academic year.

An electronic questionnaire was designed for this study. It contained items on demographics (age, college, academic level, marital status, family income, the education levels of the father and mother, and family type), anthropometric data (height (cm), weight (kg), body mass index (calculated as weight in kg divided by height in m squared, or kg/m^2^), nutritional awareness (definition of dietary supplements, their usefulness, and safety), knowledge and consumption of recommended daily servings of fruits and vegetables based on the Ministry of Health's Healthy Food Palm [28], and patterns of consumption of dietary supplements (reasons for taking supplements, where they purchase supplements, who prescribes the supplements, number of supplements actually taken, frequency of taking supplements, and the continuity of taking supplements).

All data were analyzed using SPSS Statistics 15 (SPSS Inc., Chicago, IL). The frequencies, percentages, means, and standard deviations were calculated for the variables, as appropriate, and their associations were examined via chi-square tests. The associations were considered significant if *p* values were less than 0.05. All results have been summarized in tables and graphs.

## 3. Results

Table 1 shows the demographic data of participants. Just over half (53.3%) were from the Sciences and Health Colleges, while 46.7% were from the Humanitarian and Community Service Colleges. Most participants were in academic levels 5–10 (i.e., third year to their final year; 76.6%). Most of the participants were unmarried (86.4%), and about 55.3% were from families with monthly incomes of SR 10,000 or less (about USD 2,666). For mother's and father's education, more than half of the participants had fathers with a university level education or above (51.9%), while most (64.8%) had mothers with a secondary level education or below. Most participants (74.6%) were from nuclear families (comprising the mother, father, and siblings only).

Table 2 shows participants' average height, weight, and BMI; there was no significant variation in body measurements, and their average was within the ideal weight range.

About (67.7%) took supplements occasionally and 32.3% took them regularly.

Table 3 shows the consumption patterns of dietary supplements. The proportion of participants who cited aesthetic reasons (e.g., skin or hair health) as the main reason for taking supplements was roughly equal to the proportion who cited health promotion as the main reason (45.6% and 42.4%, respectively). Participants primarily purchased supplements from pharmacies (67.9%), with only 14.5% and 3.7% purchasing supplements from electronic or social media sites, respectively. The largest proportion of participants took supplements on the advice of a doctor or dietitian (43.6%), while some reported taking them based on recommendations from friends and relatives or the advice of individuals on electronic/social media sites (26.2% and 26.5%, respectively). The proportion of participants who used more than one type of dietary supplement (39.1%), while one-third took only vitamin supplements (35.7%); only 32.6% of participants used more than one supplement at the same time. About 35.1% and 34.7% of participants took dietary supplements more than 5 times per week and less than twice per week, respectively. The majority (66.4%) of participants reported taking supplements regularly and without interruption. Over half (58.9%) of the participants claimed that they experienced benefits from using dietary supplements, and 53.4% confirmed that they would use dietary supplements when they considered this to be necessary in the future, while 35.7% reported that they would continue consuming them.

Table 4 shows the relationship between participants' demographic characteristics and dietary supplement awareness. Most (89.9%) of them correctly defined dietary supplements, and 43.6% believed that dietary supplements improve health; roughly half (53.2%) believed that they are safe. When asked about daily recommendations of the intake of fruits and vegetables, 69.3% and 67.1% of participants provided correct answers, respectively. We found that correctly defining dietary supplements was associated with the college at which participants were studying and marital status (*p* < 0.05). Marital status was also associated with dietary supplement intake, while father's educational level was associated with whether participants believed that dietary supplements improve health.

Table 5 shows the relationship between participants' intake of fruit and vegetables. We observed a statistically significant relationship between participants' consumption of fruits and vegetables, although their overall consumption of both was low.

Table 6 shows that although most participants correctly noted the recommended daily serving of fruits and vegetables, their actual consumption was typically lower. There was a negative relationship between these variables; that is, those who correctly reported the recommended daily servings were significantly more likely to report low daily consumption of fruits and vegetables.

## 4. Discussion

Nearly all the participants (89.9%) correctly defined dietary supplements, indicating that they had accurate knowledge of supplements. Furthermore, 43.6% of participants thought that taking supplements could improve their health, and 53.2% said that they considered dietary supplements safe; by contrast, 35.2% did not know the extent of their safety. Aina and Ojedokun [29] reported that about 48% of their participants were unsure of the safety of dietary supplements and their side effects. Furthermore, about 35% thought that supplements were safe, which is considerably less than the proportion in the current study. The percentages found reported by Aljaloud and Ibrahim [18] were relatively similar to those in the present study: 41.8% of their participants (professional athletes from Saudi Arabia; mean age = 25.7 ± 2.9 years) reported using dietary supplements, while 44.9% believed that supplements are sometimes safe. As for the study of female college students in Saudi Arabia [24], about 36.7% of the sample in that study had no information about the side effects of dietary supplements. Balzo et al. [30], who studied adolescents (mean age = 17,9 ± 0.9 years), found that 83.6% of participants were aware of what dietary supplements did, which is rather close to what we found. We found that large proportions of participants consumed few fruits and vegetables per day (78.4% and 49.5% respectively), even though a similarly large proportion were aware of the recommended daily intake quotas of these foods. Braun and Venter [25] also found that participants had good knowledge of the recommended daily intake of fruits and vegetables but low actual consumption: 92.0% and 47.3% knew the daily intake recommendations of fruits and vegetables, respectively, while only 65.2% and 14.3%, respectively, actually consumed the recommended amounts. Similar findings were obtained in the studies in Saudi Arabia: specifically, 78% of students of King Faisal University in Al-Ahsa ate less than 5 servings of vegetables and fruits [26], and while most students of the Faculty of Medicine at the University of Dammam knew the importance of vegetables and fruits, they did not consume them in the daily recommended quantities [27]. Thus, awareness of daily dietary recommendations of vegetables and fruits does not equate to actual consumption of these foods in the recommended quantities. The demand for supplements might therefore be a means of complementing this shortage.

More than half the participants (67.7%) sometimes took supplements whenever they believed they needed to while 32.3% had taken them for a long period. By contrast, Braun and Venter [25] found that the majority of participants used supplements (81.3%), and 79.1% used them regularly. The results of this study also agree to a certain extent with the findings of Aina and Ojedokun [29], who found that 86% of students used dietary supplements, most of whom were occasional users.

The participants reported taking supplements for mainly aesthetic reasons (45.6%), such as improving their skin and hair; this was closely followed by health improvement and increased immunity (42.4%). Braun and Venter [25] reported that the main reasons for purchasing supplements were supplementing the diet and preventing diseases; this was also found by Aina and Ojedokun [29]. In Balzo et al. [30] study, most supplement takers (both males and females) reported taking them to supplement the diet. Interestingly despite the fact that these previous studies used similarly young samples, all these studies reported that the main reason for taking supplements was to complement the diet and enhance health. By contrast, in this study, aesthetic reasons were the most common. This is perhaps because the participants were all female. Another study conducted on female college students in Saudi Arabia revealed that one of the main reasons for using supplements was to maintain health and have beautiful hair [24]. This was also found in a study among university students in Japan: the primary reason for supplement use among males was muscle building, while the primary reasons among females were aesthetics and losing weight [31].

Pharmacies were typically where participants purchased their supplements (67.9%), and the highest percentage of participants received their prescriptions from either physicians or dieticians. Aljaloud and Ibrahim [18] found that most participants purchased supplements from sports centers, having been prescribed them by the sports physicians therein. The difference between their study and the present one is likely due to the sample. Balzo et al. [30] reported that doctors ranked second after sports coaches as reasons for the female participants to be taking supplements. They also reported that pharmacies were the most frequent places to buy dietary supplements (69.0%), which is a similar proportion to that reported by the current sample. Japanese university students also primarily received supplements from the pharmacies [31], while students of the University of Dammam mentioned that they typically obtained prescriptions for supplements from a doctor [32].

Participants mainly consumed more than one type of dietary supplement (39.1%), followed by vitamins (35.7%) and then mineral salts. During the study period, participants consumed mainly one type of supplement and primarily did so more than five times per week. Similar results were obtained by Braun and Venter [25], who found that most participants took vitamins, minerals, and herbs, and by Aljaloud and Ibrahim [18] and Balzo et al. [30], who both found that the largest proportion of participants consumed vitamins; however, unlike the present study, most of the participants in Balzo et al.'s study took supplements less than twice a week. The study conducted on Japanese university students revealed that vitamin/mineral supplements were the most commonly used and that most participants used one or two types of dietary supplement (46.0% and 31.7%, respectively) [31].

This study revealed that more than two-thirds of the sample did not consume dietary supplements for more than three consecutive months, while more than half the sample reported health benefits after using them. Additionally, more than half the sample said that they would buy them in the future when they believed it to be necessary. In Aina and Ojedokun's [29] study, 84.0% of participants were willing to continue taking dietary supplements and 48.5% rated dietary supplements as very important. Owens et al. [33] found that 71.5% of participants preferred dietary supplements to traditional medicines for maintaining health. Balzo et al. [30] reported that 84.6% of their participants claimed that they benefited from using food supplements, while 79.4% said they would consume them again if they believed it to be necessary.

It is noteworthy that knowledge of the definition of dietary supplements was significantly associated with participants' college and marital status. Marital status also influenced intake of dietary supplements, while the father's level of education had a significant association with students' responses as to whether taking supplements improves health. Braun and Venter [25] found that sex, higher education, and younger age were associated with use of dietary supplements. Fattahzadeh-Ardalani et al. [34] showed that the use of dietary supplements among university students is statistically linked with social status and was more prevalent among married individuals than among unmarried ones. This is consistent with the results of the present study; that is, while most participants were unmarried, use of supplements was more prevalent among married women. This finding could be attributed to married women's interest in preserving their health and beauty; however, no studies have actually explored this relationship in detail, so the mechanism for the relationship is obscure. Furthermore, a study conducted on students in medical colleges in the eastern part of Saudi Arabia showed that the consumption of supplements was not associated with social status [23]. This might be due to differences in gender from the current study.

The present study has at least one major limitation that should be addressed before its results can be extrapolated to the general public: the sample size was too small to be representative of all 30,000 female students attending Princess Nourah Bint Abdulrahman University.

## 5. Conclusion

Nearly all (89.9%) of the participants in this study could define supplements correctly, and 43.6% believe that supplements improve health. More than half the sample stated that they believe that supplements are safe. A large proportion of participants correctly identified the recommended daily servings of fruits and vegetables, even though relatively few actually consumed the recommended amount of these foods. All participants reported having consumed dietary supplements at some point, while 32.3% were taking them at the time of the survey. They took supplements primarily for aesthetic reasons (e.g., hair and skin health) and preferred purchasing supplements at pharmacies. More than one-third of participants consumed more than one type of supplement at the same time, and more than half reported that they would use supplements when they believed it to be necessary in the future. Based on these results, it would be fruitful to conduct long-term studies on the pattern of consumption of dietary supplements among all ages and sexes and inclusive of all regions of Saudi Arabia.

## Figures and Tables

**Table 1:** Demographic characteristics of the study sample.

Demographic data	*n* (%)
*Colleges*	
Science and health colleges	405 (53.3)
Humanitarian and community service	354 (46.7)
Total	759 (100)
*Academic level*	
Basic level to level 4	178 (23.4)
Levels 5 to 10	581 (76.6)
Total	759 (100)
*Marital status*	
Single	656 (86.4)
Married	95 (12.5)
Separated or widowed	8 (1.1)
Total	759 (100)
*Income*	
SR 10,000 or less	420 (55.3)
>SR 10,000	339 (44.7)
Total	759 (100)
*Father's educational level*	
Secondary level or below	365 (48.1)
University level or above	394 (51.9)
Total	759 (100)
*Mother's educational level*	
Secondary level or below	492 (64.8)
University level or above	267 (35.2)
Total	759 (100)
*Type of family*	
Nuclear (father, mother, and siblings)	566 (74.6)
Complex (father, mother, siblings, grandfather, grandmother, etc.)	193 (25.4)
Total	759 (100)

**Table 2:** Anthropometric data for study sample.

Body measurements	M	SD
Height	158.9	7.1
Weight	59.2	13.1
BMI	23.5	5.3

**Table 3:** Consumption pattern of dietary supplements.

Dietary supplement consumption pattern	Frequency (%)
*Why do you take dietary supplements?*	
Weight reduction	59 (7.8)
Enhancing health	322 (42.4)
Increase in muscle mass	32 (4.2)
For aesthetic reasons	346 (45.6)
Total	759 (100)
*Where do you usually buy dietary supplements?*	
Pharmacies	515 (67.9)
Electronic websites	110 (14.5)
Social media sites	28 (3.7)
Stores where dietary supplements are sold	106 (13.9)
Total	759 (100)
*Who advised you to use dietary supplements?*	
Doctor or nutritionist	329 (43.4)
Scientific magazines	16 (2.1)
Friends	199 (26.2)
Websites or social media	201 (26.5)
TV ads or magazines	14 (1.8)
Total	759 (100)
*What kind of supplements do you normally consume?*	
Vitamins	271 (35.7)
Minerals	85 (11.2)
Amino acids	11 (1.5)
Fats	59 (7.8)
Dietary fiber	27 (3.7)
Herbs	9 (1.2)
More than one type	297 (39.1)
Total	759 (100)
*How many dietary supplements do you usually take at the same time?*	
One	512 (67.5)
Two	210 (27.7)
Three or more	37 (4.9)
Total	759 (100)
*How many times per week do you take dietary supplements?*	
>5	266 (35.1)
2–5	230 (30.3)
<2	263 (34.7)
Total	759 (100)
*Do you usually continuously take them for more than three months without medical advice?*	
Yes	117 (15.4)
No	504 (66.4)
Sometimes	138 (18.2)
Total	759 (100)
*Have you noticed benefits from taking supplements?*	
Yes	447 (58.9)
No	108 (14.2)
I do not know	204 (26.9)
Total	759 (100)
*Will you buy them again in the future?*	
Yes	271 (35.7)
No	83 (10.9)
When necessary	405 (53.4)
Total	759 (100)

**Table 4:** Relationships between demographic characteristics and dietary awareness (chi-square).

	College			Academic level			Marital status					
	Science and health	Humanitarian and community service	Total	Basic level to level 4	Level 5 to level 10	Total	Single	Married	Separated or widowed	Total		
*What is a dietary supplement?*												
Preparations for supplementing the diet	376	306	682 (89.9)	159	523	682 (89.9)	595	79	8	682 (89.9)		
Special diet	16	22	38 (5.0)	10	28	38 (5.0)	25	13	0	38 (5.0)		
I do not know	13	26	39 (5.1)	9	30	39 (5.1)	36	3	0	39 (5.1)		
Total	405 (53.3)	354 (46.7)	759 (100)	178 (23.5)	581 (76.6)	759 (100)	656 (86.4)	95 (12.5)	8 (1.1)	759 (100)		
*p*	0.011^*∗*^			0.912			0.001^*∗∗*^					
*Does taking dietary supplements improve health?*												
Yes	174	157	331 (43.6)	71	260	331 (43.6)	280	48	3	331 (43.6)		
No	11	13	24 (3.2)	8	16	24 (3.2)	21	2	1	24 (3.2)		
I do not know	19	18	37 (4.9)	11	26	37 (4.9)	36	1	0	37 (4.9)		
Sometimes	201	166	367 (48.4)	88	279	367 (48.4)	319	44	4	367 (48.4)		
Total	405 (53.3)	354 (46.7)	759 (100)	178 (23.5)	581 (76.6)	759 (100)	656 (86.4)	95 (12.5)	8 (1.1)	759 (100)		
*p*	0.806			0.405			0.267					
*Are dietary supplements safe?*												
Yes	207	197	404 (53.2)	90	314	404 (53.2)	340	60	4	404 (53.2)		
No	49	39	88 (11.6)	19	69	88 (11.6)	74	13	1	88 (11.6)		
I do not know	149	118	267 (35.2)	69	198	267 (35.2)	242	22	3	267 (35.2)		
Total	405 (53.3)	354 (46.7)	759 (100)	178 (23.5)	581 (76.6)	759 (100)	656 (86.4)	95 (12.5)	8 (1.1)	759 (100)		
*p*	0.458			0.514			0.141					
*According to Saudi dietary guidelines, how many daily servings of fruits should be consumed?*												
0–1	41	38	79 (10.4)	23	56	79 (10.4)	72	7	0	79 (10.4)		
2–4	285	241	526 (69.3)	120	406	526 (69.3)	450	68	8	526 (69.3)		
>4	79	75	154 (20.3)	35	119	154 (20.3)	134	20	0	154 (20.3)		
Total	405 (53.3)	354 (46.7)	759 (100)	178 (23.5)	581 (76.6)	759 (100)	656 (86.4)	95 (12.5)	8 (1.1)	759 (100)		
*p*	0.789			0.455			0.315					
*According to Saudi dietary guidelines, how many daily servings of vegetables should be consumed?*												
0–2	32	39	71 (9.4)	24	47	71 (9.4)	64	7	0	71 (9.4)		
3–5	280	229	509 (67.1)	113	396	509 (67.1)	435	67	7	509 (67.1)		
>5	93	86	179 (23.6)	41	138	179 (23.6)	157	21	1	179 (23.6)		
Total	405 (53.3)	354 (46.7)	759 (100)	178 (23.5)	581 (76.6)	759 (100)	656 (86.4)	95 (12.5)	8 (1.1)	759 (100)		
*p*	0.265			0.095			0.640					
*Do you take supplements?*												
Yes	126	119	245 (32.3)	50	195	245 (32.3)	194	48	3	245 (32.3)		
No	0	0	0 (0)	0	0	0 (0)	0	0	0	0 (0)		
Sometimes	279	235	514 (67.7)	128	386	514 (67.7)	462	47	5	514 (67.7)		
Total	405 (53.3)	354 (46.7)	759 (100)	178 (23.5)	581 (76.6)	759 (100)	656 (86.4)	95 (12.5)	8 (1.1)	759 (100)		
*p*	0.484			0.200			0.000^*∗∗*^					

	Income			Father's educational level			Mother's educational level			Type of family		
	SR 10.000 or less	>SR 10.000	Total	Secondary level or less	University level or higher	Total	Secondary level or less	University level or higher	Total	Nuclear	Complex	Total
*What is a dietary supplement?*												
Preparations for supplementing the diet	379	303	682 (89.9)	330	352	682 (89.9)	444	238	682 (89.9)	507	175	682 (89.9)
Special diet	24	14	38 (5.0)	20	18	38 (5.0)	24	14	38 (5.0)	26	12	38 (5.0)
I do not know	17	22	39 (5.1)	15	24	39 (5.1)	24	15	39 (5.1)	33	6	39 (5.1)
Total	420 (55.3)	339 (44.7)	759 (100)	365 (48.1)	394 (51.9)	759 (100)	492 (64.8)	267 (35.2)	759 (100)	566 (74.6)	193 (25.4)	759 (100)
*p*	0.564			0.127			0.945			0.240		
*Does taking dietary supplements improve health?*												
Yes	177	154	331 (43.6)	152	179	331 (43.6)	217	114	331 (43.6)	239	92	331 (43.6)
No	16	8	24 (3.2)	14	10	24 (3.2)	16	8	24 (3.2)	18	6	24 (3.2)
I do not know	22	15	37 (4.9)	15	22	37 (4.9)	23	14	37 (4.9)	28	9	37 (4.9)
Sometimes	205	162	367 (48.4)	184	174	367 (48.2)	236	131	367 (48.4)	281	86	367 (48.2)
Total	420 (55.3)	339 (44.7)	759 (100)	365 (48.1)	394 (51.9)	759 (100)	492 (64.8)	267 (35.2)	759 (100)	566 (74.6)	193 (25.4)	759 (100)
*p*	0.488			0.029^*∗*^			0.770			0.621		
*Are dietary supplements safe?*												
Yes	217	187	404 (53.2)	182	222	404 (53.2)	260	144	404 (53.2)	290	114	404 (53.2)
No	52	36	88 (11.6)	41	47	88 (11.6)	53	35	88 (11.6)	66	22	88 (11.6)
I do not know	151	116	267 (35.2)	142	125	267 (35.2)	179	88	267 (35.2)	210	57	267 (35.2)
Total	420 (55.3)	339 (44.7)	759 (100)	365 (48.1)	394 (51.9)	759 (100)	492 (64.8)	267 (35.2)	759 (100)	566 (74.6)	193 (25.4)	759 (100)
*p*	0.818			0.204			0.535			0.135		
*According to Saudi dietary guidelines, how many daily servings of fruits should be consumed?*												
0–1	40	39	79 (10.4)	39	40	79 (10.4)	53	26	79 (10.4)	56	23	79 (10.4)
2–4	292	234	526 (69.3)	244	282	526 (69.3)	340	186	526 (69.3)	397	129	526 (69.3)
>4	88	66	154 (20.3)	82	72	154 (20.3)	99	55	154 (20.3)	113	41	154 (20.3)
Total	420 (55.3)	339 (44.7)	759 (100)	365 (48.1%)	394 (51.9)	759 (100)	492 (64.8)	267 (35.9)	759 (100)	566 (74.6)	193 (25.4)	759 (100)
*p*	0.249			0.368			0.704			0.635		
*According to Saudi dietary guidelines, how many daily servings of vegetables should be consumed?*												
0–2	40	31	71 (9.4)	35	36	71 (9.4)	50	21	71 (9.4)	51	20	71 (9.4)
3–5	277	232	509 (67.1)	241	268	509 (67.1)	329	180	509 (67.1)	383	126	509 (67.1)
>5	103	76	179 (23.6)	89	90	179 (23.6)	113	66	179 (23.6)	132	47	179 (23.6)
Total	420 (55.3	339 (44.7)	759 (100)	365 (48.1)	394 (51.9)	759 (100)	492 (64.8)	267 (35.2)	759 (100)	566 (74.6)	193 (25.4)	759 (100)
*p*	0.990			0.672			0.595			0.791		
*Do you take supplements?*												
Yes	128	117	245 (32.3)	118	127	245 (32.3)	156	89	245 (32.3)	174	71	245 (32.3)
No	0	0	0 (0)	0	0	0 (0)	0	0	0 (0)	0	0	0 (0)
Sometimes	292	222	514 (67.7)	247	267	514 (67.7)	336	178	514 (67.7)	392	122	514 (67.7)
Total	420 (55.3)	339 (44.7)	759 (100)	365 (48.1)	394 (51.9)	759 (100)	492 (64.8)	267 (35.2)	759 (100)	566 (74.6)	193 (25.4)	759 (100)
*p*	0.699			0.600			0.316			0.130		

^*∗*^ *p* < 0.05^*∗∗*^*p* < 0.005.

**Table 5:** Relationship between daily intake of fruits and vegetables.

Daily intake of fruits	0-1 serving	2–4 servings	>4 servings
Daily intake of vegetables			
0–2 serving	341 (44.9)	31 (4.1)	4 (0.5)
3–5 servings	236 (31.1	96 (12.7)	7 (0.9)
>5 servings	18 (2.4)	19 (2.5)	7 (0.9)
Total and percentage	595 (78.4)	146 (19.2)	18 (2.4)
Chi-square test significance	0.00^*∗∗*^		

^*∗∗*^ *p* < 0.005.

**Table 6:** The relationship between the intake of fruits and vegetables and their recommended quantity by members of the study sample.

Daily intake of fruits	Correct answer for recommended intake		Incorrect answer for recommended intake	
	*n*	%	*n*	%
0–1 serving	434	57.2	161	21.2
2–4 servings	83	10.9	63	8.3
>4 servings	9	1.2	9	1.2
Total	526	69.3	233	30.7
*p* (chi-square test)	0.00^*∗∗*^			

Daily intake of vegetables	Correct answer for recommended intake		Incorrect answer for recommended intake	
	*n*	%	*n*	%

0–2 serving	266	35.1	110	14.5
3–5 servings	231	30.4	108	14.2
>5 servings	12	1.6	32	4.2
Total	509	67.1	250	32.9
*p* (chi-square test)	0.00^*∗∗*^			

^*∗∗*^ *p* < 0.005.

## Data Availability

The data used to support the findings of this study are available from the corresponding author upon request.
